# Current Desensitization Strategies in Heart Transplantation

**DOI:** 10.3389/fimmu.2021.702186

**Published:** 2021-08-24

**Authors:** Marlena V. Habal

**Affiliations:** Department of Medicine, Division of Cardiology, Columbia University Irving Medical Center, Columbia University, New York, NY, United States

**Keywords:** HLA, sensitization, desensitization, non-HLA antibodies, heart transplantation, cPRA, humoral immune response

## Abstract

Heart transplant candidates sensitized to HLA antigens wait longer for transplant, are at increased risk of dying while waiting, and may not be listed at all. The increasing prevalence of HLA sensitization and limitations of current desensitization strategies underscore the urgent need for a more effective approach. In addition to pregnancy, prior transplant, and transfusions, patients with end-stage heart failure are burdened with unique factors placing them at risk for HLA sensitization. These include homograft material used for congenital heart disease repair and left ventricular assist devices (LVADs). Moreover, these risks are often stacked, forming a seemingly insurmountable barrier in some cases. While desensitization protocols are typically implemented uniformly, irrespective of the mode of sensitization, the heterogeneity in success and post-transplant outcomes argues for a more tailored approach. Achieving this will require progress in our understanding of the immunobiology underlying the innate and adaptive immune response to these varied allosensitizing exposures. Further attention to B cell activation, memory, and plasma cell differentiation is required to establish methods that durably abrogate the anti-HLA antibody response before and after transplant. The contribution of non-HLA antibodies to the net state of sensitization and the potential implications for graft longevity also remain to be comprehensively defined. The aim of this review is to first bring forth select issues unique to the sensitized heart transplant candidate. The current literature on desensitization in heart transplantation will then be summarized providing context within the immune response. Building on this, newer approaches with therapeutic potential will be discussed emphasizing the importance of not only addressing the short-term pathogenic consequences of circulating HLA antibodies, but also the need to modulate alloimmune memory.

## Introduction: Sensitization to Human Leukocyte Antigens (HLA) in Heart Transplantation

HLA sensitization is a major barrier to heart transplantation, the incidence of which is increasing (1, 2). Sensitized patients wait longer for transplant and are at higher risk of dying on the waiting list or being delisted (3). Post-transplant, there is an increased incidence of adverse outcomes including both cellular and antibody mediated rejection as well as cardiac allograft vasculopathy (4–6). Heart transplant candidates bear the burden of all the same risk factors for sensitization as other transplant candidates, namely pregnancy, prior transplant, and transfusions. However, additional sensitizing events also contribute. These include homografts (7) used for congenital heart disease repair, and mechanical circulatory support (5), most commonly left ventricular assist devices (LVADs). Given that immune ‘insults’ can accumulate over time, this risk can be quickly magnified.

As is the case across solid organ transplantation, desensitization strategies are limited by variable efficacy and frequently accompanied by brisk rebound. In the case of end-stage heart disease, this is further complicated by tenuous hemodynamics, time sensitivity, and in some cases chronic device infections. In the United States, sensitized transplant candidates do not receive priority status on the waitlist (8). While policy change is one approach, prioritization can be a double-edged sword, resulting in hesitancy to develop robust, mechanistically driven desensitization strategies with the potential to improve post-transplant outcomes (9). Instead, prioritization may favor peri-transplant antibody management approaches that temporize the situation but do not modulate the underlying immune response. Multidrug regimens may more comprehensively address the underlying immune response, particularly for patients in whom years of repeated allosensitizing events result in high titer HLA antibodies with cytotoxic capabilities (10–12). However, the factors driving differences in B cell memory and plasma cell characteristics are incompletely understood, the response to treatment is heterogeneous, and therapeutic options remain limited. Moreover, while emphasis has been placed on therapeutic approaches that directly target humoral alloimmunity, it is important to consider that allosensitization includes T-cell memory and that there is growing appreciation of the complex interaction between innate and adaptive immunity. The objective of this review is to 1) summarize the current state of desensitization in heart transplantation supported by experience in kidney, 2) provide context within the immune response, and 3) building on these findings, introduce rational strategies with the potential to improve long-term outcomes. A brief overview of HLA sensitization, the methods used to detect allosensitization, and considerations unique to heart transplantation will first be provided to contextualize the review.

## Sensitization and Unique Considerations for the Heart Transplant Candidate

### Overview of the Immune Response to Foreign HLA

**Figure 1:**
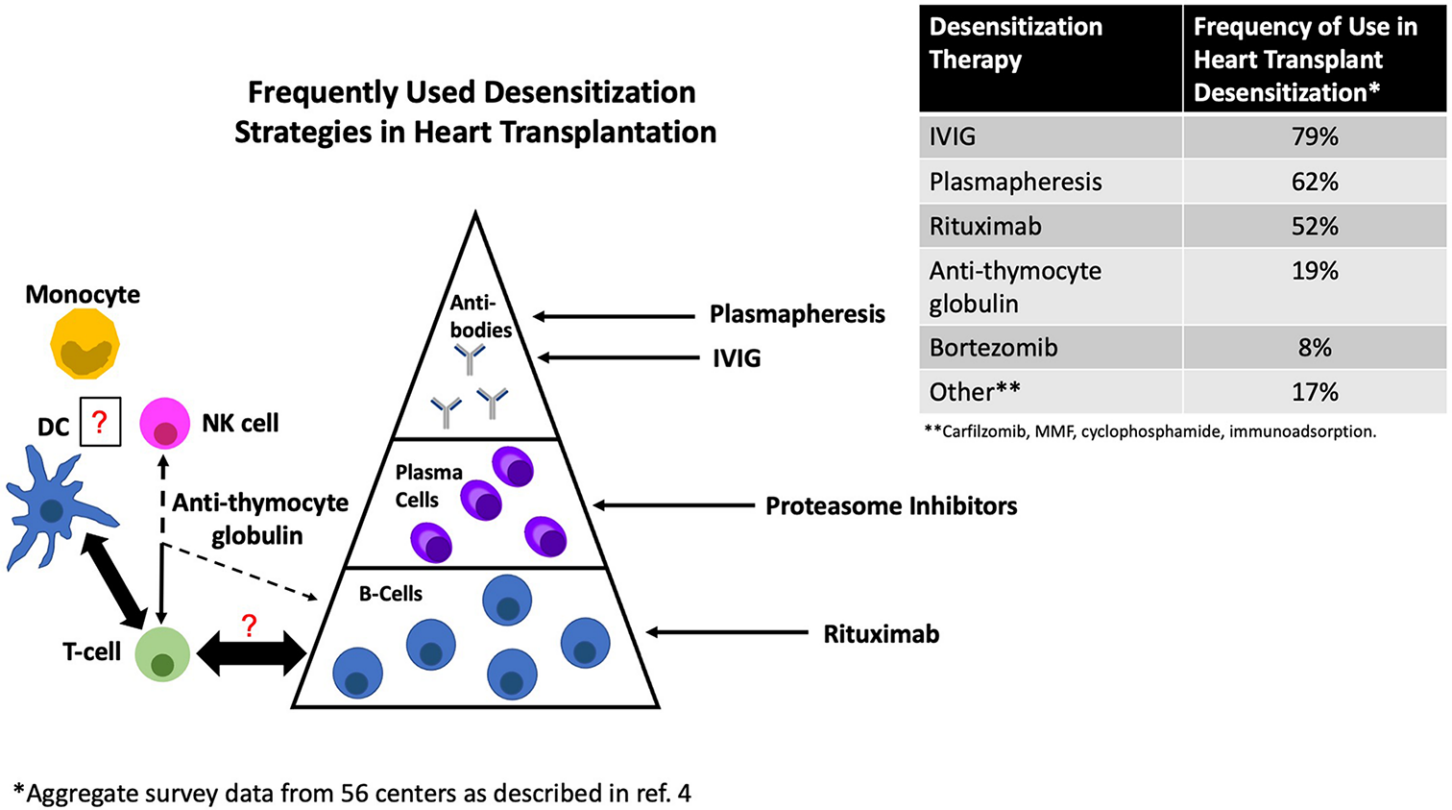
Common approaches to desensitization in heart transplantation and their primary site of action. DC, dendritic cell; NK, natural killer cell.

Exposure to foreign HLA can initiate a complex set of immune reactions which may result in a short-term ‘effector response’, long-lived plasma cells (LLPCs) capable of sustained antibody secretion over decades, and/or the establishment of B and T cell memory [reviewed in (13)]. As a result, HLA allosensitization may either be overt (detectable HLA antibodies) or cryptic (the presence of cellular memory in the absence of detectable HLA antibodies). Although only the former is commonly considered in the context of desensitization, both are associated with increased risk of rejection and/or worse outcomes after transplant (14, 15). Importantly, because HLA antibodies are the product of a T cell-dependent response (16), and because alloreactive effector T cells themselves are potently pathogenic, both T and B cell memory, as well as their ability to sustain reciprocal interactions, should be considered (17). Notably, monocytes, dendritic cells (DCs) and natural killer (NK) cells, which are at the boundary between innate and adaptive immunity, feature prominently in the response to the allograft, yet their importance in the context of desensitization is only beginning to be considered. **Figure 1** provides a schematic overview along with the most common current approaches to desensitization based on a recent international survey (4). The primary and recall humoral response are detailed in several recent reviews and summarized below (13, 18, 19). Alloreactive T cells and innate immunity will also be briefly considered.

#### The Adaptive Response to HLA Antigens: Humoral Alloimmunity

In brief, during the primary immune response, non-self HLA encountered during pregnancy (paternal), exposure to foreign tissue (transplant, homografts), or blood products (transfusions) is transported to secondary lymphoid organs (e.g., lymph nodes or spleen) which are broadly divided into B cell rich follicles and T cells zones (18). T and B cells first encounter antigen independently in their respective zones. T cells acquire a T follicular helper (Tfh) like phenotype (upregulating BCL-6, CXCR5, PD1, and ICOS) while B cells downregulate CXCR5 and upregulate CCR7 and EBI2 allowing them to migrate to the T cell-B cell border (20). Here, cognate interactions with T cells occur in the presence of costimulatory signals (CD28, CD40L, and ICOS). At this stage, B cells can either directly differentiate in an *extrafollicular manner* or enter the germinal center. Extrafollicular B cells can differentiate into antibody secreting cells (ASCs) or acquire a memory phenotype (13). Alternatively, B cells can enter the germinal center (GC) which marks another critical collaboration between T and B cells ultimately resulting in selection and clonal proliferation of B cells with a high affinity B cell receptor (BCR). Some of these may migrate to the bone marrow and become plasma cells (BMPCs), while others differentiate into quiescent memory B cells (Bmem). The understanding of these different fates and factors driving them is rapidly evolving and beyond the scope of this review. However, three concepts emerge with particular relevance from the desensitization perspective. Firstly, it is important to consider that both extrafollicular and GC B cells can acquire a memory phenotype with heterogeneity in Ig subclass, antigen affinity (resulting from somatic hypermutation), and longevity (19, 21, 22). Secondly, because BMPCs secrete antibodies with specificities that parallel those found in the blood (23), they represent an important therapeutic target. Establishing the phenotype and characteristics of anti-HLA-secreting BMPCs and the factors driving their differentiation will be critical to designing targeted therapies that ideally eliminate HLA-secreting PCs while leaving protective immunity intact. Thirdly, the opportunity for continual T cell-B cell interactions both within and outside of the GC suggests that strategies targeting these interactions may be beneficial both to dampen the active alloresponse and prevent rebound (24, 25).

#### The Adaptive Response to HLA Antigens: T-Cell Alloimmunity

Sensitized transplant candidates are at increased risk of cellular rejection highlighting the effect of enhanced T-cell reactivity towards the donor graft independent of the T-dependent antibody response (26, 27). In addition to overt exposure to antigenic HLA, the recipient’s history of encounter with environmental antigens can shape the donor reactive response through cross-reactivity or heterologous immunity (28, 29). In kidney transplant recipients, the extent of HLA molecular mismatch also influences alloimmune risk (30). For the sensitized heart transplant candidate, these factors can fine tune the donor reactive response and influence post-transplant outcomes. For the patient without an apparent sensitization history, this implies that absence of overt HLA exposure is not synonymous with low risk. At the epidemiological level, this may contribute to the heterogeneity in reported outcomes amongst HLA sensitized heart transplant recipients.

#### The Innate-Adaptive Interface in HLA Sensitization

While treatment of HLA sensitization is focused on the aforementioned adaptive immune response, innate immune cells, including monocytes, dendritic cells (DCs), and natural killer (NK) cells play an important role in the response to the allograft. DCs serve as antigen presenting cells (APCs) to activate T cells and are therefore a critical first step in the adaptive response. However, because this can occur through the direct, indirect, and semi-direct pathways [reviewed in (31–33)] activation of both CD4 and CD8 T cell can theoretically occur at any timepoint post-transplant. Monocytes are the precursors to some subsets of DCs and also macrophages the latter featuring prominently in the allograft during AMR, secreting proinflammatory cytokines, recruiting additional immune cells, present antigen locally, and provide costimulatory signals (34). NK cells are also present in the allograft during antibody mediated rejection (AMR) suggesting a logical link between DSA and graft damage *via* antibody-dependent cellular cytotoxicity (ADCC) (35). However, NK cells can function independent of DSA secreting cytotoxic, proinflammatory molecules in the setting of ‘non-self’ recognition (36, 37). While the above characterization suggests a pathogenic role for the innate response, tolerogenic potential has also been described (31, 34). Moreover, at least some innate immune cells can develop memory adding a layer of complexity to their role in the allograft over time. This suggests that manipulation, rather than inhibition of the innate immune response may be of benefit. Whether strategies that synergistically modulate innate immunity while abrogating the alloreactive adaptive response can lead to sustained improvement in long-term outcomes for highly sensitized transplant recipients, remains to be established.

### Assessment of Allosensitization

In addition to HLA antibody detection, which is most commonly done using the Luminex Single Antigen Bead (SAB) assay, numerous approaches have been developed to detect alloreactive memory T and B cells. **Table 1** provides an overview of some of these assays. It should be noted that, at the present time most of these remain exploratory in nature. For further descriptions, the reader is referred to Sensitization in Transplantation: Assessment of Risk (2017 & 2019) (38, 39) as well as several recent reviews (40, 47–49).

**Table 1:** Assays to detect & characterize allosensitization (38–46).

Assay	Description	Potential Utility for Desensitization	Limitations
**HLA antibodies**			
Luminex single-antigen bead (SAB) assay	Fluorochrome labelled beads are coated with specific HLA class I or II alleles and mixed with patient’s serum; HLA antibodies bind the bead and a secondary Phycoerythrin (PE)-conjugated anti-IgG antibody permits detection. Result reported as a normalized mean fluorescence intensity (MFI).	Permits detection and identification of the specificity of HLA reactive antibodies in the patient’s serum.	Mean fluorescence intensity (MFI) does not necessarily correlate with antibody titer.
			Inhibition, shared-epitopes, and bead saturation may affect results. This is particularly relevant in the setting of robust HLA sensitization
		Used to ‘define’ the presence of HLA sensitization.	Lack of standardization between labs in the United States.
		The most commonly used assay to assess baseline and post-treatment response. However, dilutions may be required to assess response to desensitization.	
		Post-transplant monitoring (memory *vs. de novo* response).	
Complement binding assays (e.g. C1q assay)	Same Luminex SAB assay as above. C1q binding is detected using a PE-conjugated anti-C1q secondary antibody.	Detect antibodies capable of activating the complement cascade.	C1q binding *in vitro* may not correlate with *in vivo* effects.
		Positivity may be a reflection of high titer DSA.	C1q negative antibodies can still be associated with C4d+ AMR.
		Monitor response to desensitization.	
IgG subclass	Same Luminex SAB assay as above with detection antibody that recognizes IgG1-4.	Different IgG subclasses may pose greater risk of acute rejection (e.g. IgG1 and IgG3). However, other subclasses are not without risk.	Lack of analytically validated reagents.
		Assess response of different subclasses to different desensitization strategies.	Presence of various subclasses; interpretation is limited in the context of different affinities of the reagents.
Complement Dependent Cytotoxicity (CDC) Assay	Recipient serum is mixed with donor cells (CDC crossmatch) or an established panel of cells for which the HLA specificities are known. Complement is added and cell death is quantified.	A positive CDC crossmatch is associated with hyperacute/accelerated rejection.	*In vitro* response may not be the same as *in vivo*.
			Less sensitive, more specific for cytotoxic antibodies.
			Not routinely used to determine individual antibody specificities.
**HLA reactive B cells**			
HLA tetramers by flow cytometry	Fluorochrome labelled HLA tetramers are used to detect HLA reactive B cells by flow cytometry.	Longitudinally quantify HLA reactive B cells during desensitization.	Nonspecific binding (reduced with dual fluorochrome or decoy tetramer).
		Detect differences in HLA reactive B cell phenotype in response to treatment.	Low sensitivity when limited number of B cells are present.
			Not all Bmem may be capable of secreting antibody.
			Does not quantify antibody secretion.
HLA antibody analysis in cultured supernatants following polyclonal B-cell stimulation	Polyclonal stimulation of B cells (6-day culture); detect HLA antibodies in the supernatant on the Luminex platform.	Determine magnitude of Bmem response capable of antibody secretion. Determine Bmem specificities.	Assumes all Bmem secrete the same amount of antibody. Does not quantify Bmem.
		Improve risk stratification before transplant.	Low sensitivity for low frequency Bmem.
		Implications for desensitization not well established.	Unable to characterize Bmem phenotype.
			Long culture period (6 days) makes it impractical for peri-transplant risk stratification (deceased donors).
HLA reactive B-cell ELISPOT	Polyclonal B cell stimulation (6-day culture); add HLA multimers to individual wells; detect IgG spots	More precise quantification of HLA reactive B cells with Ab secreting potential.	Need HLA multimer for each specificity of interest.
			Long culture period (6 days) makes it impractical for peri-transplant risk stratification (deceased donors).
			Labor intensive and costly.
**HLA reactive T cells**			
T-cell ELISPOT	Detect HLA reactive T cells using donor or 3^rd^ party inactivated APCs.	Quantify donor reactive T cells. Monitor changes during desensitization.	Most commonly used to measure T cells activated *via* the direct pathway of antigen presentation.
			Does not differentiate between anergy and deletion post transplantation.
			Implications in heart transplantation not well established.
Mixed Lymphocyte Reaction (MLR)	Carboxyfluorescein diacetate succinimidyl ester (CFSE) labelled recipient T cells are added to irradiated donor PBMCs; proliferation at day 6 is assessed by flow cytometry.	Assess frequency of donor reactive T cells.	Assay optimization is required to achieve reproducible results.
			Differential proliferative response between T cell subsets.
Donor reactive T cell repertoire	CFSE MLR is performed as above using pretransplant recipient T cells (responders) and irradiated donor PBMCs (stimulators). Sequencing: a. CFSE^low^ T cells are sorted (e.g. CD4 and CD8) and the T cell receptor (TCR) is sequenced. b. unstimulated recipient pretransplant T cells are sorted (CD4, CD8) and sequenced. 3. CFSE^low^ pre-transplant T-cell receptor (TCR) sequences that meet expansion criteria relative to their frequency in the unstimulated sample, are considered alloreactive. 4. Longitudinally follow alloreactive TCRs in the peripheral blood and/or biopsy specimens over time.	Pre-transplant alloreactivity can be followed prospectively during desensitization and post-transplant monitoring. Assess for clonal deletion.	As above.
			Does not account for donor reactive clones that die prior to the 6-day timepoint during the MLR.
			Detailed statistical consideration are required for interpretation.

### Unique Mechanisms of HLA Sensitization in Heart Transplantation and Their Implications for Desensitization

#### Congenital Heart Disease

Advances in surgical techniques to repair congenital heart defects have revolutionized the prognosis for children with complex congenital heart disease (CHD) with many surviving to adulthood (50–52). Some will develop end stage disease making CHD the second most common indication for heart transplantation between the ages of 18-39 (53). The risk of HLA sensitization is increased, in many cases driven by homograft material used during previous surgical repair leading to chronic exposure to foreign HLA (54). In the absence of immunosuppression brisk allosensitization ensues, which is often broadly reactive, high titer, and resistant to desensitization (7, 55–58). This is further compounded by multiple sternotomies, blood transfusions, and in some cases left ventricular assist device (LVAD) implantation (59). Given that many such patients may be turned down for transplant, the true scope of the problem is likely underestimated. Whether children, with perhaps greater immune plasticity and a shorter duration of exposure, respond differently is difficult to parse out (60–62).

#### Pregnancy & Peripartum Cardiomyopathy

Peripartum cardiomyopathy (PPCM) is an important cause of morbidity and mortality globally with a geographically polarized incidence ranging from 1:100 in the developing world to 1:2000-4000 in the US where cases may be on the rise (62–67). While many women will recover, a subset progress to end-stage disease necessitating advanced therapies. In the transplant setting, PPCM is associated with higher PRA, increased risk of rejection and worse survival (68). The divergence between the potential for a tolerogenic T cell state and the development of antibodies against paternal antigens raises important questions in the context of transplantation (69). Whether the ‘T-cell centric’ focus of our current immunosuppressive regimens, and relative lack of adequate B cell control is particularly detrimental in this setting is unknown. In a mouse model of pregnancy induced allosensitization followed by heart transplant with a semi-allogenic graft, T cell tolerance to the graft was overcome in the presence of pregnancy-sensitized B cells, whether or not DSA was present. B cell depletion (α-CD20) restored allograft acceptance (70). The clinical implications remain to be defined, but may be of particular relevance when transplant occurs in close proximity to the sensitizing event.

#### LVAD

Nearly half of patients who undergo heart transplantation are supported on an LVAD prior to transplant (71). While LVAD implant is associated with increased risk of developing HLA-reactive antibodies, their significance in terms of post-transplant outcomes is less clear (72). In an early UNOS registry analysis, LVAD recipients waited longer for transplant but there was no difference in 1-year survival or rejection episodes between sensitized and non-sensitized patients, even when highly sensitized (PRA>90%) patients were considered (72). Similarly, Chiu et al. used propensity matching to compare sensitized transplant candidates with and without mechanical circulatory support (MCS) (5). In contrast to non-MCS sensitized candidates who had increased 1-year mortality, patients transplanted from MCS had similar outcomes to non-sensitized transplant recipients.

Both the quality and duration of *de novo* detectable HLA antibody responses following LVAD implant may contribute to the discrepancy in outcomes. Using the Luminex Single Antigen Bead Assay (SAB), Shankar et al. described an increase in cPRA from 20% to 53% (p=0.024) after LVAD implant without evidence of cytotoxicity (defined as a CDC PRA>10%) (73). Similarly, a recent comparison of the HeartMate II and HeartMate 3 (HM3) device found that *de novo* sensitization, defined by SAB, persists with the newer generation HM3 although the development of high MFI (>10,000) class I antibodies was less frequent. Similar to the findings by Shankar et al, cytotoxicity defined by a *de novo* positive CDC PRA >10%, was low (74). In contrast to pregnancy and prior transplant, where high MFI HLA antibodies can persist for decades, the response post-LVAD may decline more quickly. In a cohort of 268 patients, 30 (23%) developed newly detectable HLA sensitization (defined as cPRA>10%) after LVAD implant, which declined over time in 67% of these transplant candidates (cPRA<10%) without desensitization (75). Nonetheless, compared to non-sensitized or previously sensitized transplant recipients, the risk of ACR and AMR was higher suggesting that memory persist.

Potential device-intrinsic effects on cellular and humoral immunity have been investigated (76). Whether the type of device and generation affects this is still unclear. Early studies in patients bridged to transplant with first-generation LVADs demonstrated diminished T cell responses in mixed lymphocyte reactions while non-TCR mediated activation was less affected (77). Apoptosis was enhanced, especially in the CD4 compartment although the specificity was not established. *In vivo*, the response to intradermal skin challenge with mumps and candida albicans was impaired raising the possibility of T cell dysfunction. Non-HLA humoral sensitization with reactivity to autoantigens has also been described (78, 79). In a cohort of patients bridged to transplant with a second-generation LVAD, this was attributed to natural (polyreactive) antibodies. A potential association with primary graft dysfunction but not rejection was found (80). Collectively, the increased incidence of allosensitization and predisposition to early cellular rejection raises the question of whether selective inhibition of B cell – T cell interactions and/or enhancing regulatory T cell function would be of particular benefit in this population.

### Antibodies to Non-HLA Antigens and Their Relevance to the Sensitized Heart Transplant Candidate

Antibodies to non-HLA antigens including the angiotensin II type 1 receptor (AT1R), major histocompatibility complex class I chain-related molecule A (MICA), cardiac myosin, and vimentin have been associated with increased risk of adverse outcomes in heart transplant recipients (81–86). Many of these studies were retrospective, single-center analyses and dissecting the role of pre-transplant non-HLA antibodies *versus* their development post-transplant has also been difficult. In the multicenter CTOT-5 long-term follow up cohort, the persistence at 12-months of anti-cardiac myosin antibodies (but not vimentin) present before transplant was associated with the composite endpoint of death, retransplantation, coronary stent, myocardial infarction, and cardiac allograft vasculopathy, albeit that this association was weak (87). Some but not all studies have described an association between pre-transplant anti-AT1R antibodies and increased risk of ACR, AMR, and CAV (83, 88, 89). Notably, a link between LVAD implantation and the development of anti-AT1R has also been described making this antibody of particular relevance to the heart transplant population (82). Functionally, anti-AT1R can exert agonistic effects on the AT1 receptor, induced ERK kinase signaling in endothelial cells, and promoted vascular changes in a rat kidney transplant model (90) suggesting that a complement/Fc receptor independent mechanism may contribute to its pathogenic effects.

The possibility that non-HLA antibodies synergize with DSA to exert allograft damage is salient to the highly HLA sensitized transplant candidate (83, 91). Non-HLA antibodies may either be directed towards donor polymorphisms or be reactive to autoantigens. Zhang et al, found that transplant recipients who developed anti-MICA antibodies directed at donor polymorphisms (but not against non-donor polymorphisms) were at risk of AMR (84). In a separate study, pre-transplant anti-MICA was associated with inferior kidney allograft survival (92). Thus, in already high-risk HLA sensitized heart transplant candidates, assessment of the non-HLA antibody repertoire and careful donor selection might be warranted. Conversely, in the setting of autoreactive non-HLA antibodies, donor selection would be expected to have less relevance from this perspective.

An important question is whether contemporary desensitization strategies used for HLA antibodies are effective for non-HLA sensitization. This may depend in part on their mechanism of action and the relative importance of antibody concentration/titer to downstream effect. In kidney transplant recipients with malignant AT1R antibodies, plasmapheresis and IVIG, with the addition of an ARB improved allograft survival (90). In the small subset of patients studied, anti-AT1R became undetectable. Furthermore, in highly HLA sensitized heart transplant candidates, bortezomib was associated with a reduction in anti-AT1R (93). The success of using an ARB in conjunction with plasmapheresis/IVIG ± rituximab in anti-AT1R+ heart transplant recipients with allograft dysfunction is not clear. In a recent report, although symptoms were ameliorated, only a fraction of patients experiencing improvement in graft function (94). Further work is needed to establish how anti-AT1R and other non-HLA antibodies should be managed, particularly in highly HLA sensitized heart transplant candidates.

## Current Approaches to Desensitization in Heart Transplantation

### Antibody Reduction Strategies

#### Clinical

The use of IVIG to permit transplantation amongst sensitized transplant candidates was described in the early 1990s as an extension of its observed effects in autoimmune mediated disease (95–99). Today it continues to be included in up to 79% of heart transplant desensitization protocols making it the most commonly used treatment (4). While there have been no randomized controlled trials of IVIG for desensitization in heart transplantation, its efficacy in kidney transplant was formally addressed in the randomized, placebo-controlled IG02 trial (100). Monthly IVIG infusions (2g/kg) led to a modest but significant reduction in PRA and a trend towards an increase in transplantation, albeit after 6-months when the PRA had returned to near baseline.

In a study of 13 sensitized children awaiting heart transplantation, 77% of sensitized patients defined by C1q-single antigen bead (SAB) PRA >18% were successfully desensitized with IVIG and transplanted with 100% 1-year survival (101). Similarly, in 13 children with homograft repair, those receiving higher cumulative doses of IVIG were more likely to respond to treatment although longer follow-up time was a confounding factor (60). IVIG has also been used to desensitize LVAD recipients with newly elevated CDC PRAs after implant. Dowling et al. reported on 4 LVAD recipients that developed an elevated CDC PRA after LVAD implant. All four resolved their CDC PRA to baseline within 6 months of starting IVIG (102). Similarly, John et al. described a cohort of 26 heart transplant candidates supported on an LVAD who were treated with monthly IVIG and cyclophosphamide. Compared to untreated sensitized candidates (n=45) desensitization reduced the time to transplant and decreased the risk of post-transplant rejection down to that of non-sensitized recipients (103, 104). However, these observations should be considered in the context of the aforementioned points regarding LVAD-related HLA sensitization. Indeed, the magnitude of IVIG’s effect has been challenged with the use of the more sensitive SAB assay. Nair et al, described a modest reduction in mean MFI with interindividual variability in response (105). The cPRA was not significantly reduced. A similarly heterogeneous response was observed when both the microcytotoxicity assay (CDC PRA) and Luminex SAB were performed in a small cohort of kidney transplant candidates, albeit that the dose of IVIG was lower (106).

More commonly in heart transplantation, IVIG has been used in conjunction with plasmapheresis (PP). Pisani et al. used PP and IVIG immediately prior to transplant and suggested that outcomes were similar to those of a contemporary unsensitized cohort (107). Similarly, Leech et al. found that plasmapheresis, with low dose IVIG reduced the PRA in many but not all heart transplant candidates (108). Underlying etiology and magnitude of the HLA antibody response may contribute to this heterogeneity as all non-responders in this study were women. Contemporary studies of perioperative plasmapheresis with IVIG also suggest that post-transplant rejection is common, likely because this approach does not address the underlying immune response (9, 109, 110).

Plasmapheresis has also been used as an adjunct to plasma cell therapies (discussed below) with the concept that antibody removal may decrease negative feedback inhibition on plasma cells enhancing protein production thereby sensitizing them to proteasome inhibition (PI). This effect remains to be formally evaluated but recent studies challenge this view. In an iterative trial of PI with and without intermittent plasmapheresis, both strategies led to a similar reduction in bone marrow plasma cells and circulating immunodominant HLA antibodies (10). Our preliminary work has also recently questioned the need for plasmapheresis during desensitization (12). Given i) the increased risk for infection in an already tenuous patient population, ii) challenges managing periprocedural anticoagulation, and hence iii) need for inpatient treatment, contemporary strategies may forgo plasmapheresis. Nonetheless, in very highly sensitized patients, when crossing DSA, its use in the peri-transplant setting may still be warranted.

#### Mechanistic Considerations

Multiple immunomodulatory effects have been proposed including the neutralization and enhanced elimination of pathogenic antibodies, inhibition of downstream complement activation, and direct inhibitory effects on the cellular immune response (reviewed in (97, 98, 111, 112)). From a desensitization perspective, early work proposed that IVIG effectively neutralized the pathogenic effects of anti-HLA antibodies through antiidiotypic antibodies and potentially complement inhibition (95, 113–116). The presence of anti-idiotypic antibodies to autoantibodies in IVIG preparations has been described (117). In a similar manner, Tyan et al, found that IVIG effectively inhibited cytotoxicity *in vitro*, which was attributed to the Fab2’ portion of anti-idiotypic antibodies (114, 115). It was further proposed that IVIG may stimulate endogenous production of anti-idiotypic antibodies with potential protective effects (118). While this may explain the observed reduction in CDC PRA, it has also been attributed to complement inhibition. Either way, this could explain the reduction in CDC PRA seen in at least some patients but would have lesser effect on antibody titer.

IVIG may also enhance the elimination of pathogenic antibodies by saturating endogenous Fc neonatal receptors (FcRns) (119–121). Under physiological conditions, endogenous FcRns rescue endocytosed immunoglobulin, recycling it to the cell surface thereby preventing degradation and extending its half-life in the serum. When FcRns become saturated due to supraphysiological levels of IgG, immunoglobulin is targeted for lysosomal degradation thereby reducing its half-life in the serum. Since FcRn can mediate IgG recycling at a rate 42% higher than production (122), this pathway is critical for maintaining protective immunity but may also drive the persistence of pathogenic antibodies. FcRn-/- mice eliminate IgG markedly faster than wild type, an effect which has been capitalized on to study the use of exogenous IVIG in the treatment of autoimmune disease. In an experimental model of bullous pemphigus, FcRn-deficient mice were resistant to disease and had lower levels of pathogenic IgG, an effect that could be recapitulated in disease-prone wild-type mice by the administration of high-dose IVIG (123). In time course studies, Bleeker et al, used a monoclonal antibody (mAb) to trace the effect of single-dose IVIG on antibody elimination (124). In mice this resulted in a 40% reduction with maximal effect at 3 days. Extrapolating this model to humans, a 25% reduction in autoantibody was predicted with maximal effects at 3-4 weeks although differences between species make interpretation difficult (121). Importantly, it should be noted that enhanced Ig elimination has ramifications for treatment regimens that incorporate mAb therapies with high-dose IVIG. Total dose of mAbs may be reduced or augmented depending on their Fc properties. Thus, until strategic, rigorously tested multidrug regimens are designed to specifically capitalize on these properties, administration of IVIG should be temporally separated from that of mAbs by at least 2 weeks (125).

While IVIG and therapeutic modulation of its pathway may reduce pathological antibodies in the circulation, the underlying immune response is largely unaffected. In patients treated with IVIG and plasmapheresis followed by splenectomy at the time of transplant, histological analysis suggested that immune cell composition was undisturbed (110). Moreover, examination of paired bone marrow samples obtained before and after treatment with plasmapheresis, IVIG, and anti-thymocyte globulin found no significant effect of treatment on the number of alloantibody secreting cells (109).

#### New Antibody Targeted Therapies for Highly Sensitized Transplant Candidates

Taking advantage of a potent bacterial immune evasion strategy, the IgG-degrading enzyme derived from *Streptococcus pyogenes* (IdeS) has been used for perioperative desensitization in kidney transplant recipients (126). IdeS hydrolizes IgG into the Fab2’ fragment and Fc portion thus effectively preventing antibody dependent cellular cytotoxicity (ADCC) and complement activation. Twenty-five kidney transplant candidates received IdeS 4-6 hours before transplant. All received lymphodepletion (Atgam^®^ or alemtuzumab). Some patients also received IVIG and rituximab. As expected, all circulating IgG was cleaved within 6 hours and remained inactivated for 1-2 weeks after which IgG levels increased. DSA rebound was especially notable in the absence of adjunctive B cell therapies. However, patients who received rituximab/IVIG as adjunctive therapy nonetheless had inflammation on 6-month protocol biopsies. Furthermore, infectious complications were common, and would be anticipated to be even higher/more serious amongst heart transplant recipients many of whom are supported on a LVAD as bridge to transplant. A more ‘tempered’ approach could be to enhance elimination of pathogenic antibodies by blocking FcRN, a strategy being actively studied in the setting of autoimmunity (127–130). IgG concentrations (but not IgA or IgM) are reduced by ~50% but interestingly, tetanus and influenza A reactive antibodies were preserved (127). The implications for HLA antibodies are currently being investigated. If proven, FcRN inhibition with adjunctive therapies to modulate the underlying immune response (discussed below) could reduce HLA antibodies, preserve protective immunity, and be used to suppress DSA until cellular donor reactivity is controlled.

An alternative approach is to block the downstream effects of pathogenic HLA antibodies. The complement inhibitor eculizumab has been studied in kidney transplant recipients and more recently in heart transplantation with favorable short-term outcomes (131, 132). The strength of this approach is that it permits transplantation across DSA and a positive flow-crossmatch without hyperacute rejection. However, long-term outcomes remain suboptimal. Amongst 30 sensitized kidney transplant recipients with a positive FCXM treated with eculizumab at the time of transplant, 56.7% had evidence of chronic AMR on 5-year protocol biopsy (133). Death-censored allograft survival was similar to FCXM+ controls both of which were reduced compared to those with a negative FCXM. Because this approach does not target the underlying immune response, >50% of patients in this cohort remained FCXM+ at 1-year post-transplant, albeit that the channel shift was reduced. Whether eculizumab can be used in conjunction with approaches that better target the underlying alloimmune response requires further study.

### Plasma Cell Therapies

#### Clinical

Proteasome inhibitors (PIs) induce apoptosis in response to the accumulation of misfolded proteins. The exceptionally high rate of immunoglobulin synthesis by antibody secreting cells (ASCs) underlies their susceptibility to PI-based therapies and their efficacy in treating plasma cell dyscrasias (134, 135). Bortezomib is a first-generation PI that reversibly binds the 20S proteasome. Patel et al. were amongst the first to describe its use in 7 highly sensitized heart transplant candidates all except one of whom had undergone attempted desensitization with other therapies (7 to 177 days prior) (136). Four doses of Bortezomib with corticosteroids were administered, each immediately following 2 sessions of plasmapheresis which was used in an attempt to stimulate protein synthesis by PCs. Treatment led to a marked reduction in cPRA from 62% to 35% (p=0.01) although this was determined 1-2 weeks following treatment making it difficult to differentiate between the effect of plasmapheresis, bortezomib, and prior cycles of treatment. Five patients were transplanted, 4 without evidence of rejection. One died early post-transplant in the setting of graft dysfunction and sepsis. Notably however, only one patient was transplanted against a moderate level DSA. These preliminary results were to be further tested in a multicenter, randomized-controlled trial (CTOT-13). However, this was terminated due to inadequate enrollment highlighting the challenges of these nonetheless important clinical trials. Larger studies in kidney transplant candidates suggest a modest response to bortezomib. Woodle and colleagues used an iterative study design to investigate the use of a bortezomib based protocol (with rituximab and plasmapheresis) (137). In this cohort, 43.2% of patients were successfully desensitized and transplanted, all with a negative flow crossmatch and undetectable DSA. An encouraging reduction in immunodominant antibody was observed, especially when more frequent/higher density dosing was used (8 doses). Yet the response for very high MFI antibodies was less robust. Using a cPRA MFI cut off of 8000, only 50% of patients were defined as responders and this decreased to 38.3% when the CDC PRA was used. Thus, while promising, when extrapolating to the very highly sensitized cohort, the approach remains somewhat limited. In a separate cohort of kidney transplant candidates, 32 doses of bortezomib monotherapy did not significantly reduce cPRA despite a modest reduction in unacceptable antigens (138). Whether the addition of plasmapheresis and rituximab would have improved outcomes, as was used in the study by Woodle et al, is unknown. Either way, the regimen was poorly tolerated with only 50% of candidates receiving the full treatment course without dose reduction.

More recently carfilzomib, a second-generation proteasome inhibitor that binds irreversibly to the 20S proteasome has been used for desensitization. Its superior efficacy to bortezomib for the treatment of multiple myeloma and reduced incidence of peripheral neuropathy make it an attractive alternative (139, 140). In a recent report, 9 treatment naïve heart transplant candidates underwent desensitization with plasmapheresis and carfilzomib 20mg/m^2^ followed by 2g/kg of IVIG (141). IgG-cPRA decreased from 76% to 40% (p=0.01) immediately after the last dose of carfilzomib (day 16). Six patients were transplanted, 5 across previously moderate MFI DSA which responded to desensitization (mean MFI pre/post desensitization 5360 and 2012 respectively). All patients received thymoglobulin induction. There was only 1 documented rejection during a median follow up of 35.1 months. This occurred in the patient with a C1q-PRA of 54% suggesting yet again a limitation for more highly sensitized candidates. Carfilzomib was studied in a cohort of kidney transplant candidates divided into two groups (10). A major strength of the study design was that it allowed for the effect of carfilzomib alone to be determined and directly compared to a protocol using pre-carfilzomib plasmapheresis. Both groups received 2 cycles with each cycle consisting of 6 doses of carfilzomib up to 36mg/m^2^. Group A received 3 plasmapheresis sessions after the last dose of carfilzomib while Group B had an additional plasmapheresis session added each week prior to carfilzomib. Median maximal immunodominant antibody reduction was 72.8% (Group 1, 69.8%; Group B, 80.1%) with no significant difference between treatment groups (p=0.698). Notably, this included an assessment at day 45 (before both groups received post-carfilzomib plasmapheresis) therefore demonstrating the direct effect of PI on reducing circulating HLA antibodies in the absence of plasmapheresis (Group A). However, antibody rebound was observed within 30 days of completing treatment underscoring the need for adjunctive therapies to sustain the response.

Both bortezomib and carfilzomib carry distinct, but not insignificant side-effect profiles, the former being associated with peripheral neuropathy while the latter, based on the myeloma literature, carries a theoretical risk of cardiotoxicity (140, 142). The magnitude of risk in heart transplant candidates is unknown and must be balanced with the potential benefits given the ultimate goal. Other complications include reversible acute kidney injury, thrombocytopenia, gastrointestinal side effects, and infection (143).

Collectively, these studies highlight the potential of plasma cell directed therapies as part of desensitization regimens, but this remains limited by i) inadequate response, particularly in very highly sensitized candidates, ii) antibody rebound/PI resistance, and iii) their side-effect profile.

#### Mechanistic Considerations

Bone marrow CD138+ plasma cells (PCs) secrete antibodies with specificities that mirror those in the peripheral blood underscoring their contribution to HLA sensitization (23). Bortezomib and carfilzomib reduce CD138+ bone marrow plasma cells (BMPCs) by ≥ 50%, including the HLA reactive repertoire in sensitized patients (23, 144, 145). PIs disrupt the balance between protein synthesis and destruction leading to accumulation of misfolded proteins, activation of the unfolded protein response (UPR), and apoptosis. In a mouse model of lupus, bortezomib reduces both short and long-lived BMPCs which correlated with the activation of the unfolded protein response (UPR) (146). Similar activation of the UPR has been observed in BMPCs from patients undergoing desensitization with carfilzomib (23). Nonetheless, the response is incomplete and accompanied by brisk rebound following treatment completion (10). Several factors may contribute. Firstly, some PCs may be particularly resistant to PIs owing to their tightly regulated and highly protective bone marrow niche. Indeed, protective immunity appears to be only partially affected with persistence of adequate, albeit somewhat reduced titers of tetanus and measles antibodies (145, 147, 148). Whether CD19-CD38hiCD138+ long-lived plasma cells (LLPCs), which harbor specificities for protective viral immunity also contribute to HLA specificities requires further study (149). Secondly, some PCs may develop PI resistance, potentially through β5 subunit mutations and/or upregulation of components of the immunoproteasome (23, 150). Thirdly, whether PI-induced apoptosis enhances the secretory rate of remaining PCs to maintain equilibrium remain to be established. Importantly, homeostatic proliferation in germinal centers (GCs) is thought to drive repopulation and has been elegantly demonstrated in a non-human primate model of AMR (151). This highlights the critical contribution of memory B cells, which can enter the GC leading to robust recall responses. Taken together, these observations argue for coordinated combinatorial therapies targeting not only the PC compartment but also GCs and memory B cells.

#### New Plasma Cell Therapies With Potential for Desensitization

Daratumumab is a mAb targeting CD38 which is highly expressed on the surface of plasma cells. Its success in treating myeloma (152) and more recently AL amyloidosis (153), has raised interest in its use for desensitization/AMR (154, 155). Notably, CD38 is also expressed on NK cells, which may provide additional benefit, particularly in the setting of AMR and chronic rejection (35, 36). Doberer et al. described a case of combined smoldering myeloma and DSA+ chronic active AMR treated with daratumumab (156). This report is notable for the extensive analysis of the peripheral blood, bone marrow, and allograft tissue. Consistent with the established mechanism of daratumumab, blood and bone marrow PCs were effectively depleted. This included DSA secreting BMPCs and was paralleled by the elimination of circulating DSA. NK cells were also reduced in the peripheral blood and allograft. Importantly, this was accompanied by a reduction in the molecular AMR score and microcirculatory inflammation. Similar resolution of AMR was described in another case (157). However, in contrast, class II DSA did not appear to be reduced by daratumumab. The authors suggested NK cell depletion as the mechanism underlying this apparently divergent response. While cautious enthusiasm is merited, a concern is that CD38 is also expressed on multiple suppressor cell lineages including IL-10 secreting Bregs, a subset of CD4+CD25+CD127lo Tregs with particularly potent suppressive capabilities, and myeloid-derived suppressor cells (158). In myeloma patients, this was accompanied by augmented CD8+ T effector memory responses with enhanced IFNγ secretion in response to viral antigens (158). Given the potential role of heterologous immunity and/or bystander T-cells in rejection, this latter finding is also noteworthy (28, 159). Interestingly, both of the aforementioned cases showed signs of TCMR on follow up biopsies consistent with the findings in a non-human primate model (155–157). Finally, as with PIs, daratumumab is unlikely to abolish upstream GC reactions and thus, antibody rebound may occur. Nonetheless, daratumumab, potentially in combination with additional immunomodulatory therapies may provide benefit in the pre-transplant setting. Proof-of-concept has been described with partial response in a heart transplant candidate allowing for transplantation across two previously unacceptable antigens (155). A pilot study in highly sensitized heart transplant candidates is awaited (NCT04088903).

The inability to adequately deplete the bone marrow compartment of HLA secreting PCs suggests that their tightly regulated microenvironments (i.e. ‘bone marrow niche’) may be a barrier to direct PC targeted therapies. Plerixafor inhibits CXCR4-CXCL12 interactions between PCs and bone marrow stromal cells, with the potential to release them from their niche thus raising the possibility that it could enhance PI mediated effects (160). This question is being addressed in kidney transplant desensitization with preliminary results showing promise (161, 162). Other possible approaches of interest include cytokine modulation and metabolic regulation. These have been reviewed elsewhere (160).

### B-Cell Therapies

#### Clinical

The brisk rebound after plasma cell directed therapies emphasizes the need to target B cell memory. To date, the most commonly used B cell therapy in heart transplantation is rituximab, a chimeric murine/human monoclonal IgG1 antibody directed against CD20, expressed on mature B cells. Near complete depletion of peripheral blood B cells is achieved through a combination of antibody-dependent cellular cytotoxicity (ADCC), complement-dependent cytotoxicity, and apoptosis (163).

While there are no randomized controlled trials in heart transplant desensitization, evidence from the kidney literature suggests that rituximab 1) has a modest, albeit heterogenous effect on the CDC PRA and T-cell flow crossmatch, 2) may be more effective than IVIG alone at preventing severe AMR/graft loss, and 3) has some effect on rebound but this is often incomplete (164). In an early clinical trial, Vo et al. investigated the benefit of adding rituximab (1g given on days 7 and 22) to an IVIG based desensitization protocol. This approach reduced the mean CDC PRA by 33%. However, like IG02, the PRA remained above 40% and confidence intervals were wide, consistent with a heterogeneous response (mean CDC T-cell PRA 77 ± 18% before desensitization, 44 ± 30% after desensitization) (165). Furthermore, 50% of the patients transplanted had a rejection episode, 31% of which had evidence of C4d staining suggesting that constraint of the amnestic response is incomplete. Nonetheless, the addition of rituximab may have some benefit over IVIG at tempering rebound. A trial comparing IVIG alone to IVIG + rituximab was stopped early due to AMR and 2 graft losses, both of which occurred in the placebo arm (166).

In heart transplantation, Patel et al. described the successful desensitization of 4 sensitized heart transplant candidates with rituximab and IVIG (167). The same group later reported long-term outcomes in 21 heart transplant recipients treated with IVIG and plasmapheresis, 5 of whom also received rituximab (168). Desensitization led to a reduction in antibody, albeit with a heterogeneous response, and patients were transplanted with a negative CDC crossmatch. Five-year survival and freedom from CAV were comparable to non-sensitized recipients. However, this study only considered patients who were successfully transplanted and therefore does evaluate treatment effect *per se*. Further small single-center experience in pediatric heart transplantation has also been cautiously favourable (169, 170). Schumacher et al, described a cohort of 14 heart transplant candidates of whom 8 were classified as responders and 5 were transplanted (170). Treatment with IVIG and rituximab increased the donor pool from 10% to 85% (range 2-100%) amongst responders. Rituximab was continued post-transplant with good short-term outcomes. However, despite cautious optimism based on these select, non-randomized reports, clinical experience parallels the heterogeneous and often inadequate response observed in the kidney RCTs.

Several other clinical observations are noteworthy. Firstly, as seen in CTOT-11, a trial of rituximab in non-sensitized heart transplant recipients, rituximab does not appear to prevent *de novo* DSA, a finding which has also been described in kidney transplantation (171, 172). Secondly, non-selective B cell depletion also affects B cells with regulatory/tolerogenic properties. This has been raised as a potential explanation for the increase in percent atheromatous volume (PAV) in CTOT-11, as well as the higher rate of cellular rejection in at least one rituximab study in kidney transplantation (171, 172). More recently, the ratio of IL-10/TNFα expressed by transitional B cells (T1B, CD24+++CD38+++) was found to predict T-cell mediated rejection in kidney transplant recipients (173). Perhaps most importantly, as further discussed below, B cell depletion in lymph nodes, spleen, and bone marrow is incomplete. Therefore, there is persistent potential for ongoing GC and extrafollicular antibody responses, as well as antigen presentation to T cells.

#### Mechanistic Considerations

While the CD20 antigen is widely expressed on most B cell subsets, it is absent on cells at both extremes of the B cell lineage, namely B cell precursors and antibody secreting plasmablasts/plasma cells. With respect to the former, this implies that B cell repopulation will follow depletion, as is typically seen within 12 months post-infusion (174). In terms of the latter, because bone marrow plasma cells can survive decades in protective bone marrow microenvironments, removing precursor B cells will not address HLA secreting PCs persisting in these reservoirs.

However, beyond this, rituximab incompletely eliminates B cells, and in particular, CD27+ memory B cells (Bmem), from the bone marrow, spleen, and lymph nodes (110, 175–178). Ramos and colleagues determined the effect of desensitization on splenic histology and cellular composition in five splenectomized patients who had undergone recent desensitization with rituximab/IVIG/plasmapheresis (110). Despite depletion of CD20+ and CD79+ B cells, CD27+ Bmem persisted. Similarly, CD27+IgD- switched Bmem persisted in lymph nodes from patients with rheumatoid arthritis (176). In contrast, naïve and unswitched Bmem were reduced. Clinically this is consistent with the robust recall response to influenza vaccination following rituximab monotherapy despite the near absence of circulating influenza specific Bmem in peripheral blood (179). Similarly, amongst sensitized kidney transplant recipients treated with rituximab, 34 of 39 HLA antibodies that increased after transplant were associated with epitopes shared with previous allografts or pregnancy (164).

An important implication is the persistence of lymphoid germinal center (GC)-like structures in patients treated with rituximab (180). Histological assessment is further supported by the failure to suppress LN IL-21 mRNA transcription, a surrogate for Tfh activity (74). Consistent with this, in a humanized CD20+ transgenic mouse model, GC B cells were most resistant to rituximab (181). Together, the persistence of Bmem, GCs, and LLPCs underly the limited ability of rituximab to adequately suppress established HLA antibody responses.

#### New B cell Targeting Strategies

Inebilizumab is a CD19-directed antibody recently approved for use in patients with neuromyelitis optica spectrum disorder (NMOSD) (182). Because CD19 is expressed on a subset of plasma cells, inebilizumab is an attractive target for desensitization which is being investigated in sensitized kidney transplant candidates (NCT04174677). An alternative approach is to modulate the factors required for B cell survival. To this end, BAFF (anti-B cell activating factor of the tumor necrosis alpha family) modulation has been attempted for desensitization without significant benefit. More recently, the BAFF inhibitor belimumab had modest benefit in preventing proteasome inhibitor induced rebound (183). Targeting survival factors and cytokines may be of benefit when used as part of a multidrug regimen but this requires further study.

### Costimulation Blockade Disrupts the Germinal Center and Suppresses the DSA Response

Thus far, the therapeutic strategies discussed focus on eliminating antibodies or the cells responsible for their production. The T-dependent nature of the anti-HLA antibody response suggests T cell- B cell interactions as a rationale therapeutic target. The critical players include CD28:CD80/86, CD40:CD40L, and ICOS : ICOSL and the ability to block these interactions is collectively referred to as costimulation blockade (184). The major clinical target to date has been the CD28:CD80/86 pathway. This has been achieved using the fusion protein CTLA4-Ig (abatacept) and its high affinity variant belatacept (185). CTLA4 binds to CD80/86 thereby preventing CD28 mediated signaling critical for T cell activation (signal 2). This underlies its rationale as maintenance immunosuppression in kidney transplantation with the theoretical advantage of inducing anergy in an alloantigen specific manner when T-cell receptor signaling is left undisturbed (signal 1 in the absence of signal 2). Importantly, in pre-clinical non-human primate studies, belatacept potently suppressed the primary antibody response following sheep red blood cell immunization (185) suggesting a potential advantage over contemporary T-cell focused immunosuppression. Clinically this translated into a strikingly low incidence of *de novo* DSA in phase 3 clinical trials (186).

The ability to control established immune responses with costimulation blockade has been addressed in mouse and non-human primate models which we have recently translated to the clinical setting amongst some of the most highly sensitized heart transplant candidates (cPRA>99%). Early work in mouse models from Anita Chong’s lab demonstrated that CTLA4-Ig could inhibit the memory B cell response, collapse ongoing GC B cell reactions, and halt the rise in alloantibody following heart transplantation (24, 187). While post-GC plasma cells were less well controlled, the addition of bortezomib to a continuous CTLA4-Ig regimen, but not bortezomib alone, led to sustained alloantibody suppression which mirrored the clinical response in a small cohort of kidney transplant recipients (188). Parallel studies, in non-human primates performed by Kwun/Knechtle and colleagues demonstrated that belatacept could abrogate the potent GC homeostatic proliferation associated with PI administration (25). This ‘dual targeting’ approach, tested in a rigorous model of allosensitization, decreased bone marrow plasma cells, lymph node isotype switched B cells and T follicular helper cells resulting in a reduction in DSA before transplant and improved post-transplant survival (148, 189). Moreover, a direct effect on BMPCs has been proposed given the expression of CD28 on a subset of long-lived plasma cells (190). Building on this work, we recently reported on the combination of belatacept with sequential cycles of PI therapy in four extremely highly sensitized heart transplant candidates (12). Our studies revealed a marked reduction in both class I and II HLA antibodies, including those with high MFI that were C1q positive and successful transplantation with a negative CDC crossmatch in three cases. Preliminary findings suggest that the reduction in many HLA antibodies may be sustained with ongoing monthly belatacept infusions. This is bolstered by observations in kidney transplant recipients where, compared to calcineurin inhibitors, belatacept more effectively constrained pre-existing DSA and led to a modest reduction in non-DSA (191, 192). Taken together, this highlights the relevance of rationally designed strategies that integrate clinical observations, with hypothesis driven findings, that have been specifically tested in model systems.

### Using Il-6 Inhibition to Modulate the Immune Response in Highly Sensitized Transplant Candidates

A conceivable alternative to direct immune cell targeting is to modulate the cytokine milieu. The theoretical advantage is that multiple levels of the immune response can be simultaneously targeted. Given its pleiotropic nature, IL-6 is an attractive cytokine that is important for both the innate and adaptive immune response. IL-6 supports Th1 and Th2 proliferation, promotes proinflammatory Th17 lineage commitment at the expense of Tregs, contributes to Tfh development, sustains B cell survival, and favors plasmablast differentiation (193). Inhibition of IL-6 can be achieved either by blocking the IL-6 receptor (tocilizumab) or direct neutralization (clazakizumab). Both have been used in kidney transplantation for desensitization and AMR. Tocilizumab is also being studied in non-highly sensitized heart transplant recipients early post-transplant to establish whether it can reduce the incidence of rejection, *de novo* DSA, re-transplantation and death at 1-year post transplant (NCT03644667).

Tocilizumab has been used for desensitization in two distinct cohorts of kidney transplant recipients. Vo et al. reported their experience with tocilizumab + IVIG in a phase I/II desensitization study (194). All patients had failed treatment with IVIG and rituximab. Five of 10 patients were transplanted with a reduction in their immunodominant DSA. However, the pre-treatment MFIs were <10,000 suggesting a potential limitation for patients with high titer antibodies. In a separate study of 13 highly sensitized (mean cPRA>97%) kidney transplant candidates, a more muted response was observed (195). Twelve patients had at least 1 prior transplant and in 92% the prior allograft remained *in situ* (off immunosuppression). Efficacy assessment was evaluated based on the immunodominant MFI, which in most cases was >10,000. In this cohort, tocilizumab monotherapy had only a marginal effect on HLA antibodies as assessed by MFI with dilutions used when prozone was detected. While an increase in naïve B cells and decrease in plasmablasts was observed, tocilizumab did not significantly change the percentage of pre/post-germinal center B cells, Tfh, and Tfh subsets between baseline and 6 months and there was no significant augmentation of CD3+CD4+CD127-FoxP3+ Tregs. Nonetheless, Treg augmentation with tocilizumab has been reported by others (196, 197). Subsequently, Vo et al, described the use of clazakizumab in combination with plasma exchange and IVIG for desensitization (198). A reduction in class I and II HLA antibody MFI was observed although there was substantial variability in response between patients. Alemtuzumab was used for induction along with standard immunosuppression and clazakizumab was continued post-transplant. Notably, only 1 patient had detectable DSA at 12 months raising the possibility that adjunctive post-transplant IL-6 blockade may be of benefit.

A potential role for IL-6 inhibition has also been described in the setting of AMR. Choi et al, reported on a cohort of 36 kidney transplant recipients with cAMR treated with tocilizumab after failing standard therapies (199). A significant reduction in DSA, C4d deposition, glomerulitis and peritubular capillaritis scores was observed. Graft survival was 80% at 6-years which is higher than that reported in the literature without treatment although a control arm was not included. A subsequent study randomized 20 patients with DSA positive late AMR to clazakizumab *vs.* placebo followed by an open label extension where all participants received study drug (200). Clazakizumab reduced DSA with improvements in intragraft AMR associated findings in some patients during the extended follow up period. Early attenuation of eGFR decline was observed in the clazakizumab arm compared with placebo which was no longer apparent when the placebo arm was transitioned to clazakizumab. Serious infection (n=5) and diverticular disease with complications (n=2) were noted suggesting the need for careful pre-treatment evaluation and close monitoring.

Collectively, these studies suggest that IL-6 inhibition may be of benefit to the allosensitized transplant patient both before and after transplant. The full potential of IL-6 inhibition together with adjunctive strategies for the highly sensitized candidate remains to be determined. Conceptually, synergism between IL-6 inhibition and costimulation blockade could augment GC inhibition while counterbalancing the potential reduction in Tregs. Other combinations are also possible and future studies, perhaps driven by a translational approach, should be considered.

### Considering Cell-Based Therapies for the Allosensitized Heart Transplant Candidate

A potential alternative or complementary strategy to the ones described thus far is to modulate the immune response using cell-based therapies such as mesenchymal stromal cells (MSCs) or regulatory immune cells (e.g. Tregs, dendritic cells, macrophages). This approach remains largely speculative for desensitization and a full discussion of the topic is beyond the scope of this review. However, several pre-clinical and conceptual considerations are noteworthy. MSCs have been shown to inhibit alloantibody production *in vitro (* 201), reduce DSA in a rat model of transfusion associated allosensitization (202), and increase rejection-free survival in a high-risk corneal transplant model (203). Clinical translation was reported in three HLA sensitized patients (204) although cautious interpretation is warranted given the need to address potential risks of MSC-based therapy. Firstly, the use of allogeneic MSCs has been associated with the development of low-level DSA to the MSC and/or shared kidney-HLA (205). This was prevented when repeat mismatches (MSC and allograft) were avoided (206). Secondly, MSCs have been associated with transient kidney graft dysfunction when given in the immediate post-transplant period (207). This was attributed to early post-transplant inflammation resulting in MSC recruitment to the allograft where they potentiated the proinflammatory response. MSC administration prior to transplant may prevent this. Nonetheless, the possible implications for the heart transplant recipient are noteworthy given that hemodynamic compromise can be fatal. Thirdly, the risk of opportunistic infection and malignancy requires ongoing evaluation (208). Nonetheless, in addition to the aforementioned humoral alloimmune effects, MSCs may modulate multiple layers of the immune response enhancing T-cell suppression, augmenting transitional/regulatory B cells, modulating dendritic cells and macrophage activity (reviewed in (208, 209)). The potential to use 3^rd^ party MSCs in the pre-transplant setting would significantly enhance feasibility in heart transplantation where the timing of transplant is not *known a priori.*

An alternative strategy is to use Tregs to suppress the anti-donor response, ideally in an antigen specific manner. This could theoretically target alloreactive effector T cells while leaving infectious immunity intact. Safety has been preliminarily established in phase I/II trials including those in the multicenter ONE Study (210, 211). However, these trials were done in low-to-intermediate risk subjects with the goal of demonstrating safety and/or permitting immunosuppression minimization. Less is known about the effect on humoral alloimmunity and more saliently, the relevance to pre-transplant allosensitization. In a mouse model of AMR, induced Tregs reduced DSA and allograft infiltration/damage in some, but not all animals (212). More recently, Sicard et al, investigated the effect of donor specific CAR-Tregs (dsCAR-Tregs) on the humoral response in a mouse allogeneic skin transplant model (213). Compared to controls, dsCAR-Tregs reduced the number of DSA secreting B cells, decreased the amount of *de novo* DSA, and prolonged survival. In contrast, when mice were sensitized prior to transplant, dsCAR-Tregs were neither able to constrain the humoral alloimmune response nor prolong survival suggesting that this approach may not be as effective for sensitized transplant candidates. Whether combining Tregs with adjunctive strategies such as B cell depletion would be of benefit, requires further study. However, practical issues (such as the unpredictability of timing in heart transplantation) and safety implications (including the stability of Treg phenotype) are important to consider in this setting.

## Discussion: Challenges and Future Directions

In this review the literature relevant to heart transplant desensitization has been summarized, supported by finding at the cellular level in human studies, and objective rationale derived from model systems. Interpretation is limited by 1) a paucity of standardized clinical trials in heart transplantation, 2) ethical limitations in assigning a control arm, 3) immense variability in the etiology and ‘degree’ of sensitization, 4) lack of standardization across HLA labs, and 5) deficits in our understanding of the factors driving memory B cell and plasma cell differentiation, persistence, and resistance to desensitization. Given that humoral sensitization rates have doubled over the past 2 decades (4) and that highly sensitized candidates are more likely to die waiting for transplant or become too sick (3), developing safe and effective desensitization strategies is an urgent priority.

One aspect that has as yet not been addressed in this review is the sensitization ‘cut-off’ meriting desensitization. This remains a challenging issue. Using cPRA based on MFI can be problematic. Not only is MFI an inadequate surrogate for antibody titer, but the choice of MFI cut-off will determine whether the candidate is desensitized thus introducing heterogeneity. Attesting to the variability in practice, in a recent international survey, 21% of centers used cPRA>80% and 21% used cPRA>50% as their cut off. The remainder described using a range of cut-offs from >10% to >90% (4). The next important consideration is whether the desensitization strategy will alter long-term outcomes. Approaches that have the potential to modulate the underlying donor reactive immune phenotype and/or repertoire, as discussed in this review, may be of benefit even when sensitization is more modest. This has yet to be formally evaluated. However, at present, the most commonly used approaches for desensitization are plasmapheresis, IVIG, and rituximab (4). Neither alone nor in combination are these likely to suffice, particularly in the very highly sensitized candidate and the potential for durable effects is modest.

**Figure 2:**
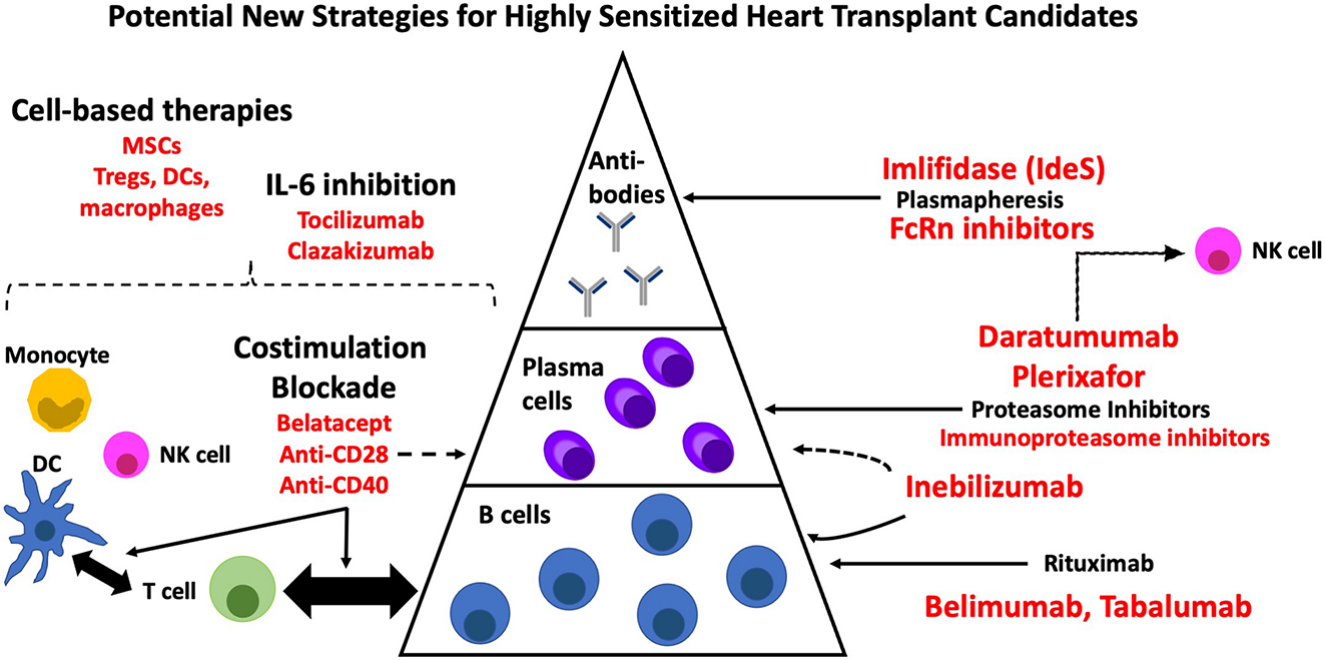
New therapies with potential for desensitization in heart transplantation. DC, dendritic cell; MSC, mesenchymal stromal cell; NK, natural killer cell.

Developing a prioritization policy, as has been implemented in Canada (9), may be of value but is limited by the lack of standardization across HLA labs in the US, differences in what constitutes an unacceptable antigen, and difficulties defining an optimal cut-off. Furthermore, this approach may foster hesitancy to pursue robust immunomodulatory desensitization strategies with the potential to improve long-term outcomes. Whether some patients, perhaps those who are more advanced in age, may be better served by destination LVAD is another consideration. However, this is not an acceptable option in many cases where heart transplantation remains the gold-standard. The focus should therefore be on designing multidrug regimens, driven by drug repurposing, and developed in conjunction with findings from pre-clinical models. While the present review has put forth several strategies with potential to target each layer contributing to the immune response (**Figure 2**), other approaches are forthcoming. Improvements in our understanding of HLA reactive memory B cells and plasma cell characteristics is critical to defining who will derive the greatest benefit from targeted therapies. Post-transplant immunosuppression with better control of B cell memory, ideally in an antigen specific manner, will also be required. Long-term benefit will likely be derived from strategies that modulate multiple layers of the immune response. Despite inroads being made, much remains to be done to ensure equal access to heart transplantation for the highly sensitized patient with end-stage heart disease. While desensitization continues to be a ‘niche’ field, the growing incidence in an already high-risk population along with the poor prognosis for those denied transplant underscores the urgent need for attention.

## Author Contributions

The author confirms being the sole contributor of this work and has approved it for publication.

## Funding

Mendez National Institute of Transplantation Foundation (PG012633).

## Conflict of Interest

The author declares that the research was conducted in the absence of any commercial or financial relationships that could be construed as a potential conflict of interest.

## Publisher’s Note

All claims expressed in this article are solely those of the authors and do not necessarily represent those of their affiliated organizations, or those of the publisher, the editors and the reviewers. Any product that may be evaluated in this article, or claim that may be made by its manufacturer, is not guaranteed or endorsed by the publisher.

## Abbreviations

ACR, Acute Cellular Rejection; ADCC, Antibody-Dependent Cellular Cytotoxicity; AMR, Antibody Mediated Rejection; AT1R - angiotensin II type 1 receptor; Bmem, Memory B cell; BMPC, Bone Marrow Plasma Cell; CDC, Complement Dependent Cytotoxicity; CHD, Congenital Heart Disease; cPRA, calculated Panel Reactive Antibody; CTLA4, Cytotoxic T-Lymphocyte-Associated Protein 4; DSA, Donor Specific Antibody; FcRN, Neonatal Fc Receptor; FCXM, Flow cytometric crossmatch; GC, Germinal Center; HLA, Human Leukocyte Antigen; IdeS - IgG-degrading enzyme derived from *Streptococcus pyogenes*; IVIG, Intravenous Immunoglobulin; LLPC, Long Lived Plasma Cell; LN, Lymph Node; LVAD, Left Ventricular Assist Device; MCS, Mechanical Circulatory Support; MFI, Mean Fluorescence Intensity; MICA, Major Histocompatibility Complex Class I Chain-Related Molecule A; PI, Proteasome Inhibitor; PPCM, Peripartum Cardiomyopathy; PRA, Panel Reactive Antibody; SAB, Single Antigen Bead; TCR, T-cell Receptor; UPR, Unfolded Protein Response.
